# Enhancement of in vitro antitumour activity of epirubicin in HER2+ breast cancer cells using immunoliposome formulation

**DOI:** 10.1049/nbt2.12012

**Published:** 2021-02-07

**Authors:** Farnaz Khaleseh, Abbas Hemmati Azandaryani, Fatemeh Fathian Kolahkaj, Mozafar Khazaei, Katayoun Derakhshandeh

**Affiliations:** ^1^ Nano Drug Delivery Research Center Kermanshah University of Medical Sciences Kermanshah Iran; ^2^ Fertility and Infertility Research Center Kermanshah University of Medical Sciences Kermanshah Iran; ^3^ Department of Pharmaceutics School of Pharmacy Hamadan University of Medical Sciences Hamadan Iran

## Abstract

Epirubicin (EPI) is one of the potent breast cancer (BC) chemotherapeutic agents, but its adverse effects limit its efficacy. Herein, EPI was selected to be loaded in liposomal carrier, which has been targeted by a monoclonal antibody, Herceptin. The preparation process of liposomes was a modified ethanol injection method followed by Herceptin conjugation. The in vitro cell toxicity and cellular uptake of optimum formulation against HER2+ and HER2− cancer cell lines were evaluated. The results showed that the drug loading (DL%) and encapsulation efficiency (EE%) of liposome preparation method yielded 30.62% ± 0.49% and 62.39% ± 8.75%, respectively. The average size of naked liposomes (EPI‐Lipo) and immunoliposomes (EPI‐Lipo‐mAb) was 234 ± 9.86 and 257.26 ± 6.25 nm, with a relatively monodisperse distribution, which was confirmed by SEM micrographs. The release kinetic followed Higuchi model for both naked and immunoliposomes. In vitro cytotoxicity study on three different BC cell lines including BT‐20, MDA‐MB‐453 and MCF‐7 demonstrated higher toxicity of EPI in the Herceptin conjugated form (EPI‐Lipo‐mAb) in comparison with the free EPI and EPI‐Lipo in HER2 overexpressing cell line. In addition, the cellular uptake study showed a higher uptake of immunoliposomes by MCF‐7 cells in comparison with naked liposomes. In conclusion, these data show that the targeted delivery of EPI to breast cancer cells can be achieved by EPI‐Lipo‐mAb in vitro, and this strategy could be used for breast cancer therapy with further studies.

## 1 INTRODUCTION

One of the common malignancies, especially among women, is breast cancer (BC). Despite different therapeutic strategies, BC has the highest mortality rate after lung cancer [1]. More than 90% of this kind of cancer is non‐metastatic at early stages of disease [2]; therefore, site‐specific treatment decreases the risk of disease relapse. Different treatment protocols like surgery, radiotherapy or chemotherapy can be used according to the tumour and patient conditions [3]. Among them, neoadjuvant chemotherapy has shown an acceptable outcome, since it results in tumour shrinkage [4]. The treatment response rate has increased by neoadjuvant chemotherapy consisting of trastuzumab, anthracycline, cyclophosphamide and paclitaxel [5]. On the other hand, targeted drug delivery systems can improve treatment efficacy and therapeutic index. One of the most interesting agents for targeted therapy is antibodies or antibody fragments as they are cytotoxic and have the cell killing potential in addition to addressing therapeutic agent to tumour site [6]. Overexpression of human epidermal growth factor receptor 2 (HER2) in 25%–30% of BCs occurs and can be a target receptor for the antibody trastuzumab (Herceptin™) [7]. This monoclonal antibody (mAb) has shown an enhanced survival rate in the cases of HER2‐positive BCs by down‐regulation of the receptor expression. The mechanism is receptor endocytosis acceleration and cell progression hindrance by inducing p27Kip1/Cdk2 complexes and suppression of angiogenesis [8]. Hence, trastuzumab‐functionalized nanoparticles including polymeric nanoparticles, gold nanoparticles and liposomes have improved the drug efficacy of drug‐loaded nanoparticles [9, 10, 11]. Among them, immunoliposomes have high similarity to cell membrane composition and improve the pharmacokinetic parameters of the encapsulated drug because of the bilayer structure of liposome membrane; the other properties are drug release control, biocompatibility and drug accumulation in the tumour site [12]. Such a drug delivery system can be a good choice for encapsulation of anthracycline agents.

Anthracyclines exist in many cytotoxic regimens for the treatment of several malignancies such as sarcoma, BC and lymphoma as they diminish disease relapse and improve survival rate [13]. Epirubicin (EPI), an anthracycline agent, is a doxorubicin analogue with higher cell membrane permeability and high efficacy in BC, non‐Hodgkin's lymphomas, ovarian cancer, soft‐tissue sarcomas etc. [14]. EPI exists in different BC treatment protocols; although myelosuppression is an acute dose‐limiting toxicity of drug in addition to nausea and vomiting, alopecia and transient cardiac arrhythmias [15]. So the adverse effects will be controlled by encapsulation along with pharmacokinetics and antitumour efficacy improvement [16].

Herein, EPI was encapsulated in the novel immunoliposomes consisting of 1,2‐dioleoyl‐sn‐glycero‐3‐phosphoethanolamine (DOPE) and cholesterol (Chol), and was targeted for HER2 receptors by Herceptin functionalization. Liposomes were prepared by ethanol injection method followed by extrusion and mAb conjugation. Immunoliposomes were optimized according to physicochemical properties, cellular uptake was assessed and, finally, cytotoxicity was studied for the optimized formulation in three different BC cell lines to investigate the in vitro tumour cell cytotoxicity.

## 2 MATERIALS AND METHODS

### 2.1 Materials

DOPE was purchased from Lipoid AG. Chol, N‐hydroxycarbidiimide (NHS), 3‐(4,5‐cimethylthiazol‐z‐yl)‐2,5‐diphenyltetrazolium bromide (MTT), 1‐ethyl‐3‐(3‐dimethyl aminopropyl) carbodiimide (EDC), trypsin‐EDTA, were obtained from Merck. Potassium dihydrogen phosphate, dimethyl sulfoxide (DMSO), analytical grade ethanol and acetone were purchased from Sigma‐Aldrich Co. Trastuzumab (*M*_w_ 145.5 kDa) was supplied by LuyePharma. Epirubicin was purchased from Sigma‐Aldrich Co.

### 2.2 Preparation of EPI loaded immunoliposomes

#### 2.2.1 Preparation of liposomes

Liposomes were fabricated according to ethanol injection method with some modifications. Briefly, an exact quantity of DOPE and Chol (Table 1) were dissolved in an exact volume of ethanol by sonication. EPI was dissolved in the aqueous phase with different volumes. The ethanolic solution of lipid and Chol was added to the aqueous phase by syringe rapidly and with constant rate; then it was stirred for 15 min at room temperature. Liposomes were formed by evaporation of the ethanol residuals. The vesicles were three times prefiltered through polycarbonate membrane with defined pores of 0.8 micron using extruder. To allow the formation of smaller vesicles (∼200 nm), this was followed by extrusions through double‐stacked membranes with 0.2 micron pore size. During all the extrusions, the temperature was maintained at least 10°C above the glass transition temperature of the DOPE. The prepared liposomes (EPI‐Lipo) were dialysed through a membrane with cut‐off 3000 KDa to separate free EPI from the encapsulated drug [17].

**TABLE 1:** Composition, size and PDI of the EPI‐nanoliposome formulations

Formulation (*n* = 5)	DOPE (mg)	Cholesterol (mg)	Aqueous phase (ml)	Ethanol phase (ml)	Size (nm)	PDI
1	5	5	5	1	565.2 ± 10.32	0.895
2	5	5	5	2.5	406.8 ± 6.58	0.317
3	5	5	5	5	413.5 ± 11.23	0.267
4	10	5	5	1.5	351.2 ± 12.57	0.303
5	10	5	2.5	2.5	331.8 ± 13.81	0.255
6	10	5	10	2.5	322.5 ± 8.97	0.415
7	10	2.5	5	2.5	357.5 ± 11.22	0.144
8	10	6.5	5	2.5	234 ± 9.86	0.22
9	10	7.5	5	2.5	284.9 ± 15.86	0.388
10	10	7.5	5	2.5	947.8 ± 8.86	0.268

Abbreviations: DOPE, 1,2‐dioleoyl‐sn‐glycero‐3‐phosphoethanolamine; EPI, epirubicin; PDI, poly dispersity index.

#### 2.2.2 Monoclonal antibody conjugation

Amide bond formation is the basis of trastuzumab conjugation. So, for the preparation of functionalized nanoliposomes, the mAb should be activated with EDC and NHS. NHS ester creates reactive acylating agents with nucleophiles such as primary amine and releases its leaving group to form a stable amide bond. The addition of aminated antibody to prepared liposomes was performed under N_2_ and shaken at room temperature for 2 h for direct coupling of mAb on liposome surface. Immunoliposomes were dialysed through a membrane with 12,000 cut‐off for 2 h to remove the unreacted antibody. Finally, the solution was lyophilized and a fine powder of Herceptin conjugated liposome (EPI‐Lipo‐mAb) was obtained and stored at 4°C for further analysis (ZibrusVaco 10‐II‐E; Germany) [18].

**FIGURE 1:**
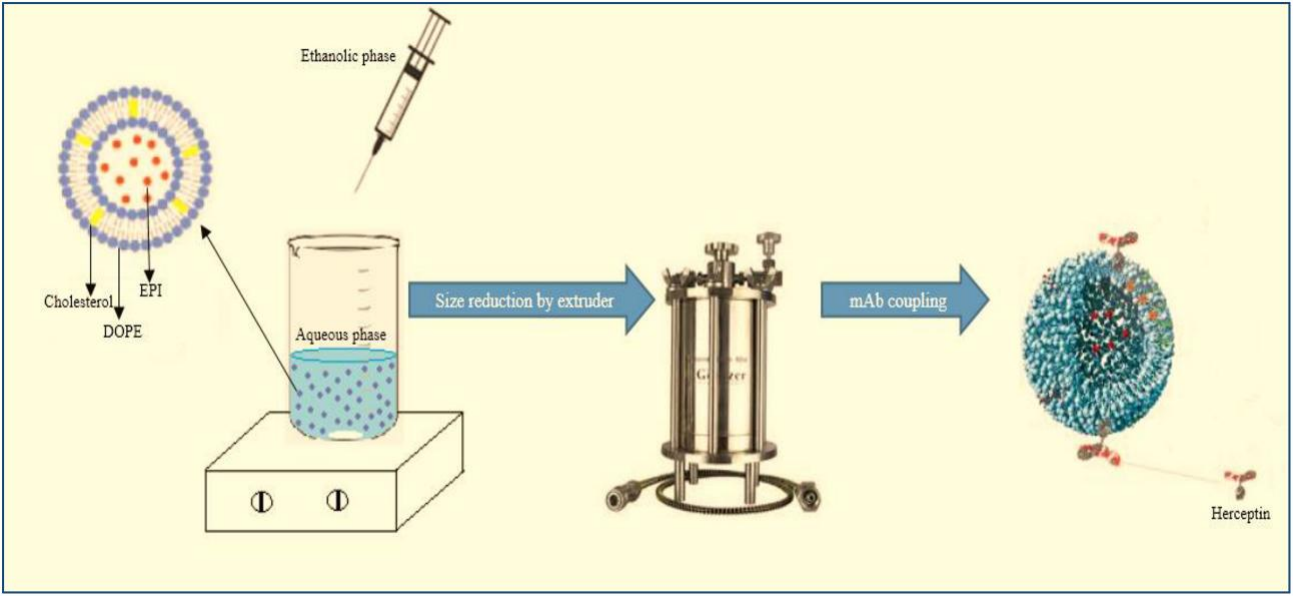
Schematic preparation method of naked and immunoliposomes. DOPE, 1,2‐dioleoyl‐sn‐glycero‐3‐phosphoethanolamine; EPI, epirubicin; mAb, monoclonal antibody

Figure 1 shows the schematic preparation method of naked and immunoliposomes.

#### 2.2.3 Monoclonal antibody coupling assay

The Bradford protein assay is a spectroscopic analytical procedure used to measure the concentration of protein in a solution. For this aim, 180 μl of Coomassie dye plus reagent was added to an ambient amount of free dispersion of mAb, and after 10 min of incubation, the absorbance was measured at 595 nm using a UV‐Visible instrument (Schimadzu). The results were compared to a standard curve of mAb solution in the concentration range of 15–60 μg/ml [9].

### 2.3 Determination of drug loading and encapsulation efficiency

The percentage of incorporated EPI was calculated indirectly and has been reported as drug loading (DL) [19] and encapsulation efficiency (EE) [20] according to Equations (1) and (2). For this calculation, after the separation of free drug by dialysis bag, the amount of EPI in the outer medium of dialysis bag was measured by UV‐Visible spectrophotometer at 255 nm (Schimadzu) and EE% and DL% were calculated as follows:

(1) $$\text{EE}\left(\%\right)=\left(W_{a}-W_{s}\right)/W_{a}\times 100$$

(2) $$\text{DL}\left(\%\right)=\left(W_{a}-W_{s}\right)/\left(W_{a}+W_{s}+W_{\text{DOPE,Ch}}\right)\times 100$$

where *W*_*a*_, *W*_*s*_, and *W*_DOPE_,_Ch_ were the weight of the added drug, the analysed weight of the drug in the dialysis medium, and the weight of DOPE and cholesterol added to prepare liposomes, respectively.

### 2.4 Physicochemical characterization of immunoliposomes

#### 2.4.1 Morphology, particle size and zeta potential determination

The samples (naked liposomes and immunoliposomes) were diluted with distilled water, and after drying for 12 h, they were placed on an aluminium pan and coated with a gold thin layer. The scan was conducted at an accelerating voltage of 10 kV by capability of point‐to‐point scanning by XL30 microscope (Philips, the Netherlands) for scanning electron microscopy (SEM) images [19].

The particle size, poly dispersity index (PDI) and zeta potential of the naked liposomes and immunoliposomes were measured by dynamic light scattering (DLS) (Malvern Zetasizer ZS). The vesicles were diluted with distilled water (ratio of 1:3) and measurements were conducted at room temperature.

#### 2.4.2 Fourier transform‐infrared spectroscopy spectra analysis

Fourier transform‐infrared spectroscopy (FT‐IR) spectra of samples were studied by Schimadzu IR‐prestige 21 FTIR spectrometer to find the sample interactions. Therefore, the evaluated samples were naked and immunoliposomes, free mAb, Chol, DOPE and EPI. Each sample tablet was prepared by mixing 2 mg of sample with 10 mg KBr followed by compression. Transition mode was applied for sample evaluation and the spectral region was 450–4000 cm^−1^.

#### 2.4.3 Differential scanning calorimetry

Thermal characteristics of the samples including naked and functionalized liposomes, free mAb, cholesterol, DOPE, EPI and physical mixture were characterized by differential scanning calorimeter (Shimadzu DSC‐60, single heating ramp) at the temperature range of 20–375°C and heating rate of 10°C/min. Reference was An empty aluminium pan was used as a reference, and dry nitrogen was used as effluent gas during the experiment [21].

### 2.5 In vitro drug release evaluation

Franz diffusion cell was selected to evaluate the in vitro drug release profile of naked liposomes and immunoliposomes [22]. The release medium was 25 ml phosphate buffer (0.1 M, pH 7.4) placed in the receptor part of the cell and stirred (400 rpm) at 37°C. Five milligrams of each sample was suspended in phosphate buffer and poured into the donor part of the cell. The cut‐off of separating membrane of donor and acceptor parts was 12 kDa. At distinct time intervals, 1 ml of the medium was replaced with fresh medium to establish sink condition. Samples were analysed by UV‐Visible spectrophotometer at 255 nm to find the amount of drug release. The procedure was repeated three times for each sample. The cumulative percentage of drug release versus time was plotted.

Different kinetic models were evaluated to find the best model fitted to in vitro release data. The analysis was done according to correlation coefficient (*R* ^2^) of the models given in Table 2.

**TABLE 2:** Results of release kinetics according to various kinetic models

Formulation	Zero‐order *R* ^2^	First‐order *R* ^2^	Higuchi *R* ^2^	Hixon‐crowell *R* ^2^	Weibull *R* ^2^	Korsmeyer‐Peppas *R* ^2^
EPI‐Lipo	0.951	0.962	0.997	0.966	0.938	0.945
EPI‐Lipo‐mAb	0.742	0.889	0.988	0.921	0.928	0.953

Abbreviation: EPI, epirubicin.

### 2.6 In vitro cytotoxicity investigation

The cytotoxicity assay was studied in three different cell lines containing MCF‐7, MDA‐MB‐453, and BT‐20; the method was based on MTT assay, which shows the viable cells. For this purpose, the cells were cultured in 96‐well plates (Nalgen Nunc International); the cell density was 1.5 × 10^4^ cells/well followed by 24‐h incubation for sufficient adhesion of cells. The different formulations containing different concentrations of free EPI, phospholipid, naked EPI‐loaded liposomes and EPI‐loaded immunoliposomes were added to wells and incubated for 24 h; each sample was repeated six times. Then the samples were replaced with 200 μl fresh Dolbecco's modified eagle's medium followed by 20‐min incubation. The medium was removed, then 200 μl RPMI medium and 20 μl of MTT dye solution (10% in phosphate buffer pH = 7.4) were added to each well and incubated for 2.5 h (at 37°C and 5% CO_2_). Finally, the medium was removed, and formazan crystals were solubilized by 200 μl of DMSO; the reacted dye was dissolved and the absorbance was measured at 570 nm by a microplate reader (Bio‐Tek, ELX 800).

The cell viability (%) was calculated by (*A*)_test_/(*A*)_control_ × 100, where (*A*)_test_ is the absorbance of each test sample and (*A*)_control_ is the absorbance of control sample. The culture medium without cells was considered as control, and the IC_50_ of samples (the concentration which inhibits the growth of 50% of cells) was calculated by Sigma plot software [22].

### 2.7 Cellular uptake of immunoliposomes

MCF7 cells were cultured on a 24‐well plate followed by 24‐h incubation; the initial density was 5 × 10^4^ cells/well. Then 1 µg/ml of naked and immunoliposomes were added at different time intervals to investigate the cellular uptake. Finally, the cells were washed with phosphate buffer solution three times to remove the formulations that are not taken by cells; fluorescence microscopy was used for cellular uptake investigation [23].

## 3 RESULTS AND DISCUSSION

### 3.1 Formulation physicochemical characterization

EPI‐loaded liposomes were prepared by a modified ethanol injection method. Table 1 reports different formulations with the related size and PDI. The results show that F8 is the best formulation with a minimum average size of 234 ± 9.86 nm and acceptable PDI of 0.22 ± 0.05, which shows the mono‐dispersity of liposome size. The F8 formulation composition is 6.5 mg Chol, 10 mg DOPE, 1 mg EPI with 1:2 ratio of organic to aqueous phase. As the optimum formulation, EE% and DL% of F8 formulation were 62.39 ± 8.75% and 30.62 ± 0.49, respectively. After the mAb conjugation, the size of EPI‐Lipo‐mAb was 257.26 ± 6.25 nm and that of PDI was 0.25 ± 0.08. The size of immunoliposomes is larger than naked liposomes, because of the mAb conjugation to liposome surface.

One important factor for stability investigation is the surface charge of particles. As reported in previous studies by Derakhshandeh and co‐workers, higher negative charge of particles next to more stable formulation might increase the circulation time of particles [22, 24]. The zeta potential of naked and immunoliposomes were −4.34 and −11.0 mV, respectively, which can show the high stable formulations. Very high negative surface charge may cause low interaction between drug delivery system and cell membrane more than stability [25]. Therefore, this is an acceptable surface charge for immunoliposomes.

**FIGURE 2:**
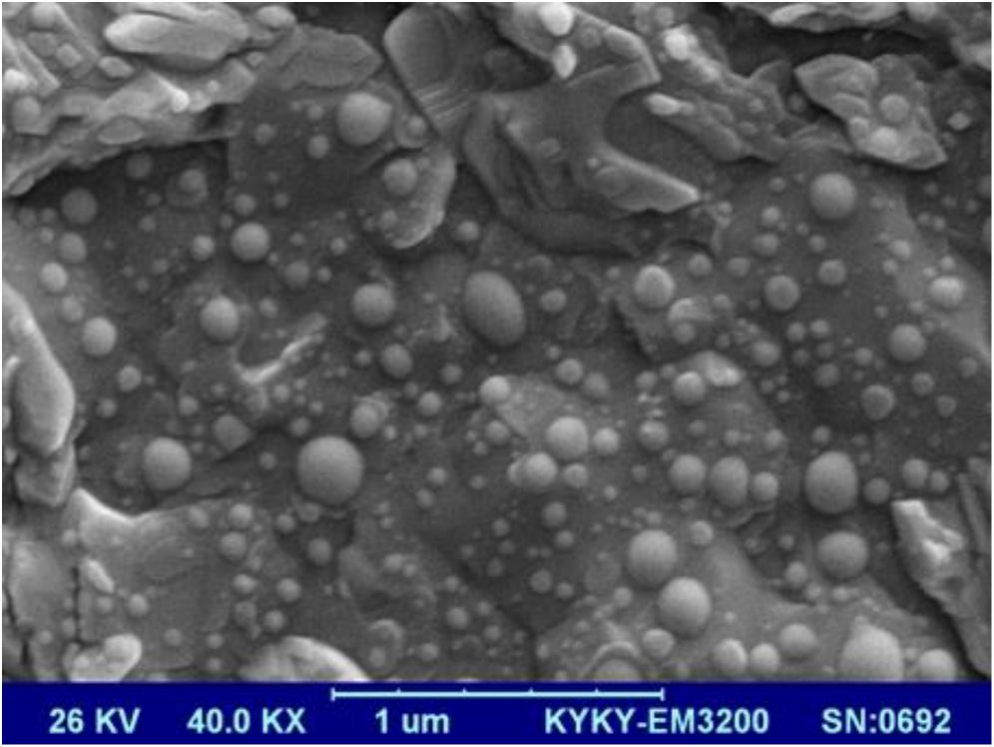
Scanned electron micrograph of epirubicin‐loaded liposomes

The spherical shape and smooth surface of particles can be observed in SEM micrographs (Figure 2). The particle size depicted in Figure 2 is in agreement with the DLS size analysis result.

### 3.2 FT‐IR studies

**FIGURE 3:**
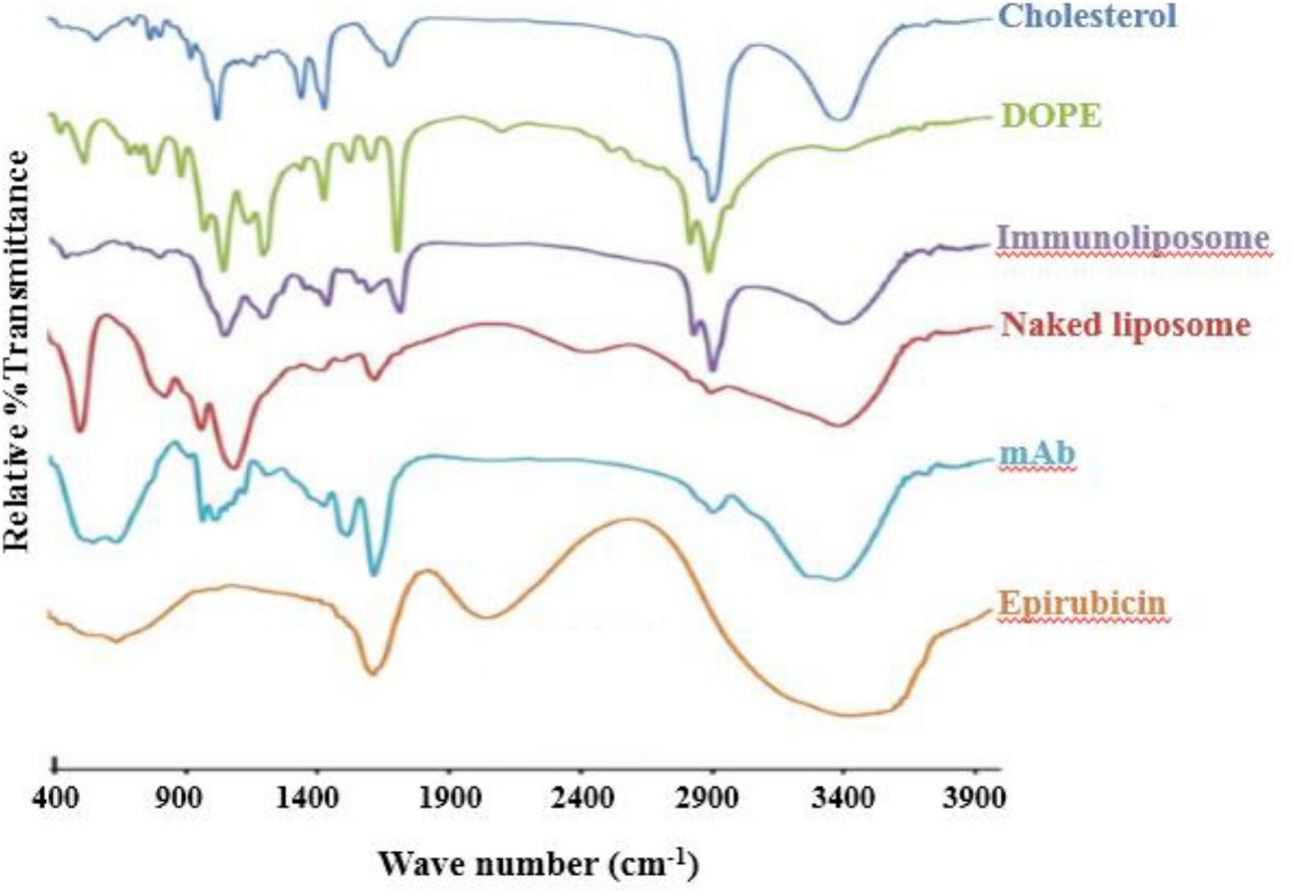
Fourier transform‐infrared spectra of different components and formulations. DOPE, 1,2‐dioleoyl‐sn‐glycero‐3‐phosphoethanolamine; mAb, monoclonal antibody

The principle goal of nanoparticle preparation is the higher DL% in the carrier; comparison of FT‐IR spectrum of final formulation and the initial components can help to find the interactions that may be formed between drug and carrier. Figure 3 shows the FT‐IR spectrum of samples. Results show that the peaks around 3500 and 1600 cm^−1^ for EPI are related to OH and NH_2_ and ketone group between two aromatic rings in the structure. There is different functional groups in the DOPE structure as it has been confirmed by the spectrum like the peak around 800 and 1700 cm^−1^ that show P‐O and ester groups. The CH3 groups' peak and double bonds in the chol structure are appeared in the spectrum around 2900 and 1600 cm^−1^, respectively. The antibody spectrum confirms the existence of two kind of amine and carbonyl group around 3300, 600 and 1600 cm^−1^. The spectrum of chol, DOPE and EPI should be found in the naked liposomes and EPI and antibody in the mAb conjugated liposomes. According to the explanations of the component spectra, the spectra around 2900, 1700, 1000 cm^−1^ confirm the chol, DOPE and EPI in naked liposomes and 3400 and 1100 cm^−1^ confirm the antibody in the immunoliposomes.

### 3.3 DSC studies

**FIGURE 4:**
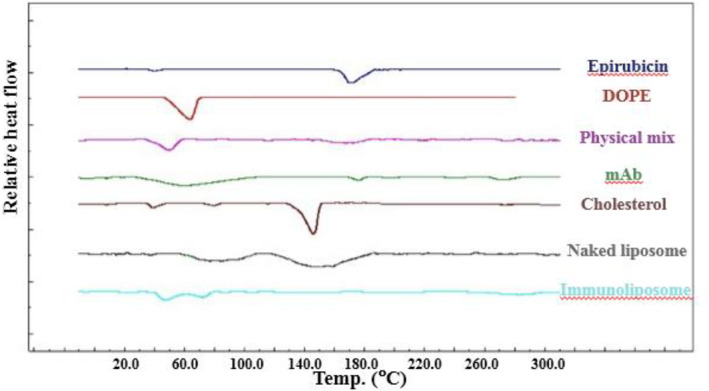
Differential scanning calorimetry thermograms of different components and formulations. DOPE, 1,2‐dioleoyl‐sn‐glycero‐3‐phosphoethanolamine; mAb, monoclonal antibody

DSC thermogram of samples is shown in Figure 4. A sharp melting point has been appeared in the EPI powder at 170.92°C with a melting enthalpy of −98.89 J/g and onset temperature of 165.70°C that also exists in the physical mixture thermogram around 175.71°C. However, no sharp melting point can be observed in two final formulations, which confirms existence of no crystalline form of EPI in the liposomes; also, the entire drug has been encapsulated in the carrier. The antibody thermogram shows a melting region with a peak of 60.32°C and melting enthalpy of −221.62 J/g and onset temperature of 31.94°C; it confirms that the antibody structure is not crystalline. The peak of antibody can be observed in immunoliposomes that confirms the surface conjugation of antibody with liposomes.

### 3.4 In vitro drug release study

**FIGURE 5:**
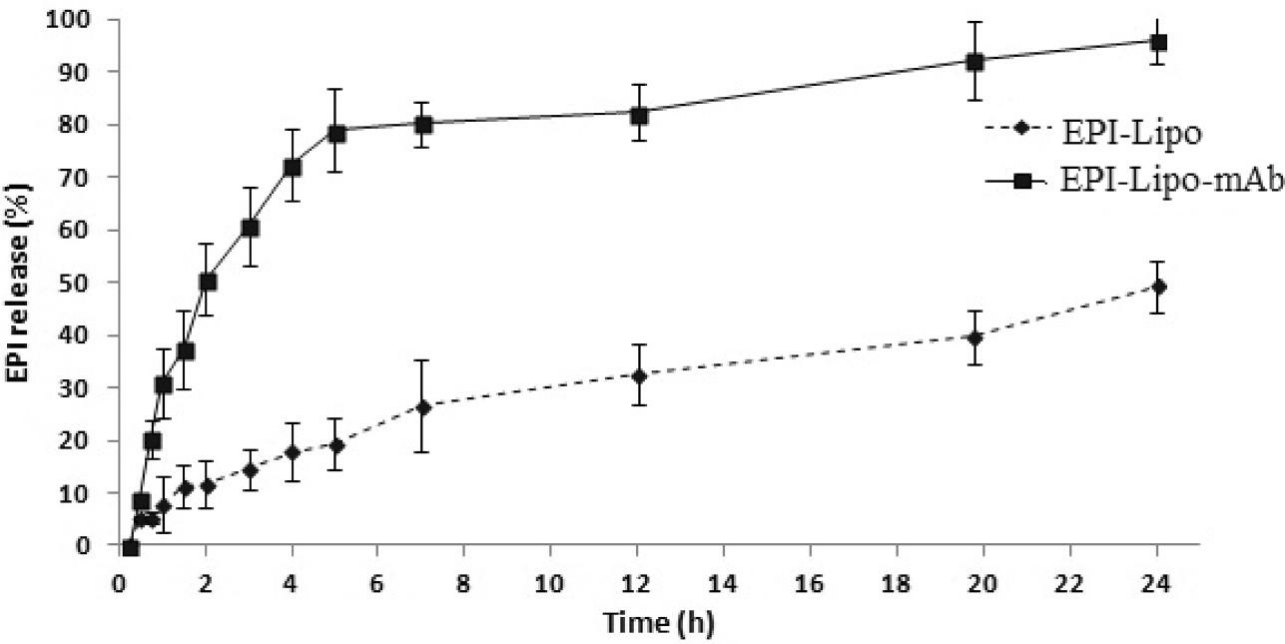
In vitro release profile of EPI from naked and immunoliposomes in PBS (pH = 7.4), the data represent mean ± SD (*n* = 3). EPI, epirubicin. PBS, phosphate buffer solution

One of the important factors for drug release is particle size by the way that smaller particles lead to higher surface area so the drug exposure for dissolution will increase and higher amount of drug will be dissolved in the medium [26]. Figure 5 shows the drug release profile of both final formulations. The cumulative drug release for naked liposomes and immunoliposomes during 24 h is about 50% and 90%, respectively. Different mathematical models containing Higuchi, Hixson‐Crowell, Korsmeyer‐Peppas, first and zero order were evaluated to find the release mechanism; so the kinetics was evaluated according to correlation values (*R* ^2^). According to the results of Table 2, the best fitted model for both formulations was Higuchi model; it confirms the diffusion mechanism of drug release by the square root of the time. Also, a higher cumulative drug release from immunoliposomes can lead to higher cellular uptake of EPI from immunoliposomes compared to naked liposomes.

### 3.5 In vitro cell line study for cytotoxicity and cellular uptake evaluation

**FIGURE 6:**
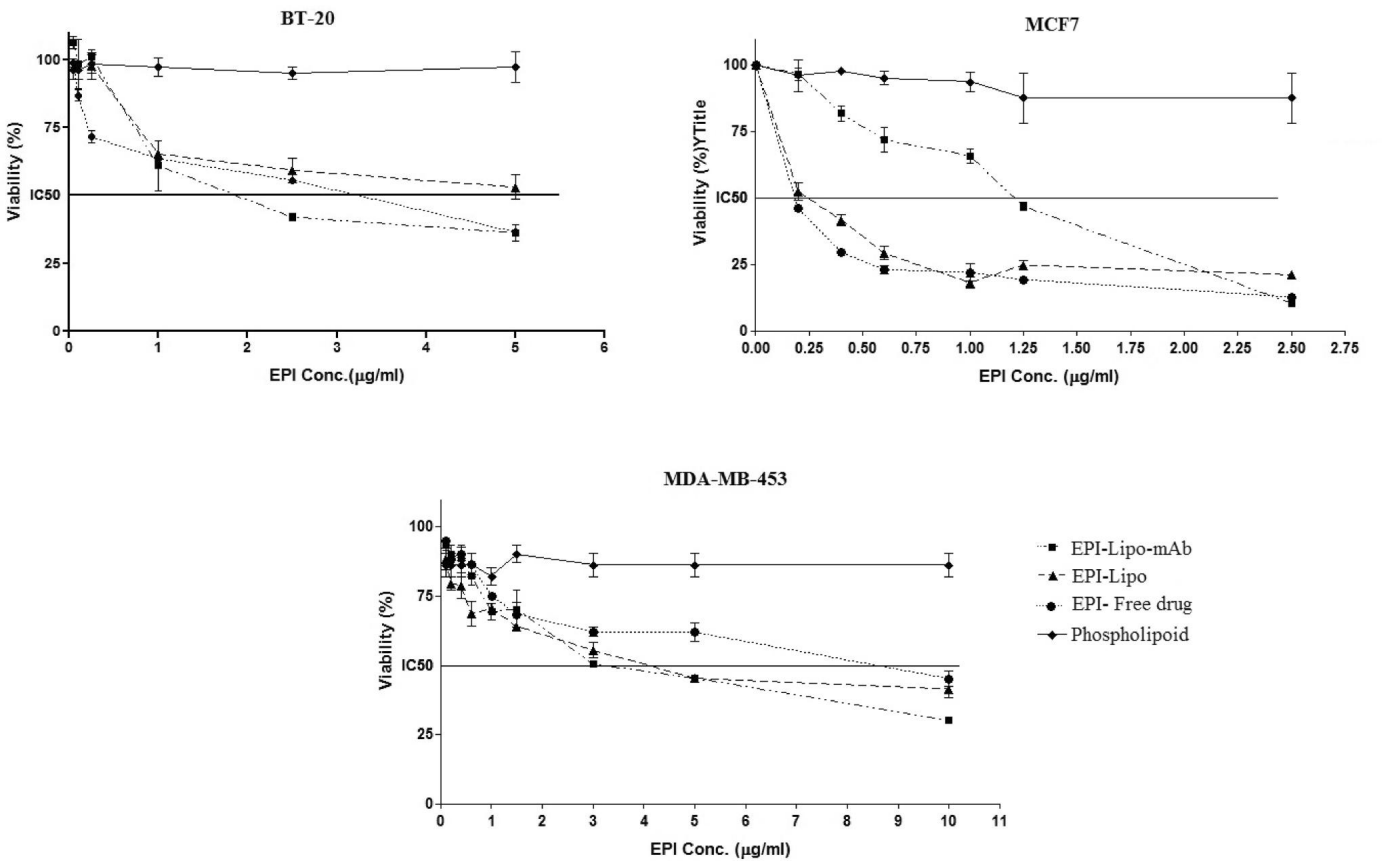
In vitro cell line cytotoxicity of different formulations, the data represent mean ± SD (*n* = 6). EPI, epirubicin

MTT assay was selected for in vitro cytotoxicity investigation of formulations. MCF‐7, MDA‐MB‐453 and BT‐20 cell lines were exposed to different concentration of formulations. The viable cells were counted and IC_50_ of each formulation was evaluated; the results are depicted in Figure 6.

It can be concluded that the phospholipid (DOPE) did not show any cytotoxicity effect on none of the cell lines in the treated concentrations.

Evaluation of the average IC_50_ of EPI in three formulations (free EPI, naked and immunoliposomes), as tabulated in Table 3, shows that IC_50_ of EPI in MCF‐7 cells is higher for immunoliposomes compared to naked liposomes. As this cell line is HER2−, the immunoliposomes could not attach to cell surface as much as to cause lower IC_50_ and so higher potency. On the contrary, the IC_50_ of EPI in BT‐20 cell line is much lower for immunoliposomes than naked liposomes. The IC_50_ of EPI in MDA‐MB‐453 is much lower even than the difference of BT‐20 for immunoliposomes in comparison with naked liposomes. The reason can be the overexpression of HER2 for MDA‐MB‐453 cells that leads to higher efficacy of loaded EPI in mAb coupled liposomes in this cell line.

**TABLE 3:** IC_50_ values of EPI formulations in different cancer cell lines (*n* = 6)

Formulation	MCF 7	BT‐20	MDA‐MB‐453
EPI free drug	0.26 ± 0.05	3.31 ± 0.21	8.41 ± 1.02
EPI‐Lipo	0.45 ± 0.08	5.58 ± 0.38	4.01 ± 0.59
EPI‐Lipo‐mAb	1.33 ± 0.28	1.98 ± 0.18	2.87 ± 0.33

Abbreviation: EPI, epirubicin, mAb, monoclonal antibody.

Comparison of the effect of immunoliposomes in three cell lines shows that EPI is more effective in MCF‐7 and BT‐20 than MDA‐MB‐453 and confirms by lower IC_50_, which can be related to the ER+ and PR+ characteristic of MCF‐7 cell line. The overexpression of ER and PR on the cell surface can lead to higher affinity of immunoliposomes [27]. The results show that the best effect of mAb coupling has been caused in MDA‐MB‐453 as it is a HER2+ cell line and the IC_50_ has been reduced to about a third of the free drug.

**FIGURE 7:**
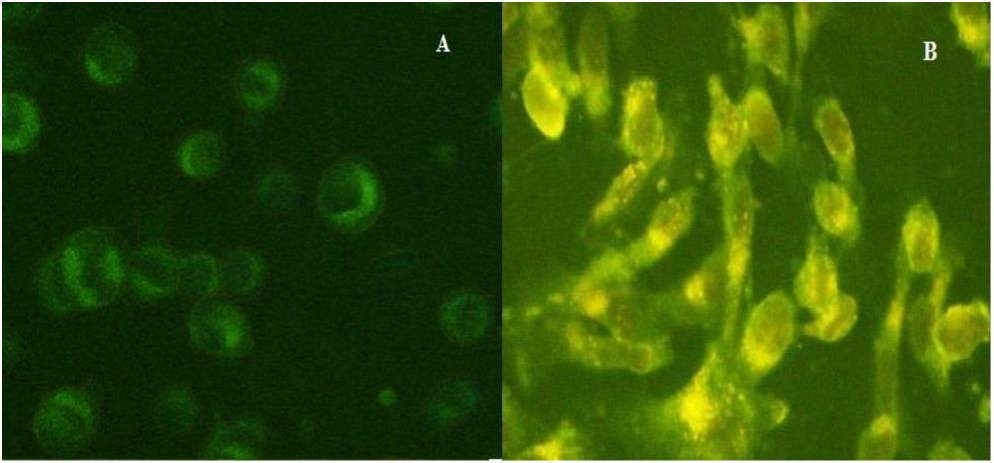
Intracellular uptake of epirubicin (EPI): (a) naked liposomes (EPI‐Lipo) and (b) immunoliposomes (EPI‐Lipo‐mAb)

Cellular uptake of naked and immunoliposomes to MCF‐7 cells was studied by fluorescence microscopy. There was no need for addition of a fluorescent dye, due to EPI fluorescence property. The amount of cellular uptake of EPI increased along with higher exposure time of formulation to cells but the fluorescence intensity was high enough to be detected even after four hrs of exposure. As shown in Figure 7, the colour intensity is much higher after immunoliposomes exposure compared to naked liposomes, which confirms the higher affinity of immunoliposomes to cells and so higher uptake of EPI.

## 4 DISCUSSION

In the described experiments, EPI‐loaded liposomes and HER2‐targeted liposomes were prepared, and after the physicochemical characteristic evaluations, these formulations were assessed by in vitro cytotoxicity and cellular uptake study. As the liposomes were targeted for HER2 receptors by Herceptin, the drug efficacy was higher than free EPI in HER2 overexpressing cells. Therefore, this kind of EPI‐loaded drug delivery system can be a potent carrier for HER2+ BCs.

Higher efficacy of immunoliposomes can be due to cytostasis/cytolysis which is caused by the carrier binding to HER2 receptors as was elucidated by Vaidya et al. They prepared dual functionalized immunoliposomes by trastuzumab (anti‐HER2) and OKT‐3 (anti‐CD3) antibodies to study the doxorubicin efficacy. The results showed high cytotoxicity for the functionalized formulations, especially when effector cells exist. They demonstrated that the mechanism can be cytostasis/cytolysis in addition to slow drug release from functionalized liposomes [28].

The mechanism of EPI uptake in HER2+ cell lines can be through receptor‐mediated endocytosis as reported by Amin et al. in the study of mAb conjugated liposomes containing idarubicin in HER2+ BC cell lines. It has been observed that trastuzumab conjugated liposomes cause more efficient attachment of drug delivery to overexpressed HER2 cell lines and so higher cellular uptake will happen [29].

Yan Wu et al. studied the EPI pharmacokinetic parameters in animal model after encapsulating in thermosensitive liposomes. The results showed that liposomes can increase the circulation time of EPI, and higher drug exposure will lead to higher antitumour activity [16].

Rodallec et al. studied the efficacy and tumour cell uptake of targeted liposomes containing Docetaxel for HER2+ BC model in vitro and in vivo. They found that immunoliposomes caused higher efficacy than free drug and antibody. They related this effect to better drug delivery because despite the higher tumour internalization, the localization of immunoliposomes was not higher for immunoliposomes, even in HER2+ cells. The results show that the EPR effect is as potent as antibody conjugation in drug targeting [30].

## 5 CONCLUSION

In the present study, Herceptin conjugated EPI‐loaded liposomes were prepared and the physicochemical properties were evaluated. EPI was successfully encapsulated which was confirmed by high EE%. The negative surface charge of particles leads to high stability of formulation and a good choice for intravenous injection. The Higuchi release kinetic shows that drug will be released continuously during 24 h. Different formulations based on various aqueous‐to‐organic phase ratio and components were prepared. The optimum formulation according to size and size distribution was selected and in vitro cellular uptake and cytotoxicity studies showed that Herceptin conjugated liposomes have higher cell toxicity in HER2+ cell lines compared to naked liposomes. Therefore, Herceptin decorated liposomes can improve EPI efficacy in HER2+ BCs. *Further* in vivo analysis should be conducted to confirm the results.

## CONFLICT OF INTERESTS

The authors declare no conflict of interest.
